# Community rehabilitation of disabled with a focus on blind persons: Indian perspective

**DOI:** 10.4103/0301-4738.60086

**Published:** 2010

**Authors:** R Jose, Sandeep Sachdeva

**Affiliations:** Directorate General of Health Services, Ministry of Health and Family Welfare, Government of India, Nirman Bhawan, New Delhi - 110 108, India

**Keywords:** Assistive devices, blindness, disability, low vision aids, program, rehabilitation

## Abstract

India, the largest democratic country in the world, is marching ahead strongly on the growth and developmental front and is poised to be the leader in the market economy. This role creates and increases far greater responsibilities on us in ensuring that the benefit of the developmental cycle reaches each and every citizen of this country, including the able and the disabled ones. It has been enshrined in the Constitution of India to ensure equality, freedom, justice, and dignity of all individuals and implicitly mandates an inclusive society. With increase in consideration of quality parameters in all spheres of life including availability, access, and provision of comprehensive services to the disabled, it is pertinent to have a look on the contribution of government in keeping the aspiration and commitment towards common people. The article attempts to review the concept of rehabilitation for the disabled keeping a focus on the blind person, and list out the activities, programs/schemes, institutional structure and initiatives taken by the Government of India (GOI) for the same and the incentives/benefits extended to blind persons. The article concludes by reiterating the importance of individual need assessment and mentioning new initiatives proposed on Low Vision services in the approved 11^th^ plan under National Programme for Control of Blindness (NPCB). The source of information has been annual reports, notification and the approved 11^th^ five-year plan of GOI, articles published with key words like rehabilitation, disability, assistive devices, low vision aids, and/or blind person through the mode of Internet. Annexure provides a list of selected institutions in the country offering Low Vision services compiled from various sources through personal communication and an approved list of training institutes under NPCB, GOI offering Low Vision training.

There has been an evolutionary process in changing attitudes regarding the disabled and blindness around the globe. About 1,000 years back the attitude was of disregard, rejection, isolation, and abuse, however, 100 years back the attitude changed to pity and benevolence. Currently, there is a positive attitude towards the disabled including the blind[1] and this becomes more important considering the fact that country incurs a huge expenditure directly in supporting and indirectly through loss of economic productivity of the disabled. This article attempts to review the concept of rehabilitation for the disabled keeping a focus on the blind, and list out the activities, programs/schemes, institutional and legal structure, and initiatives taken by the Government of India (GOI) for the same and the incentives/benefits extended to blind persons. The article concludes by reiterating the importance of need assessment and mentioning new initiatives proposed on Low Vision services in the approved 11^th^ five-year plan under the National Programme for Control of Blindness (NPCB). The source of information has been annual reports, notification and approved 11^th^ five-year plan (2007-12) of the GOI, articles published with key words like rehabilitation, disability, assistive devices, low vision aids, and/or blind person through the mode of Internet. Annexure provides a list of selected institutions in the country offering Low Vision services compiled from various sources through personal communication and/or an approved list of training institutes under NPCB, GOI offering Low Vision training.

## Concept of disability and rehabilitation[2–4]

The goal of medicine is to promote, preserve, and restore health when it is impaired and to minimize suffering and distress. These goals are embodied in the word prevention. Successful prevention depends upon knowledge of causation, dynamics of transmission, identification of risk factors and risk groups, availability of prophylactic or early detection and treatment measures, an organization for applying these measure to appropriate persons or groups and continuous evaluation and development of procedures applied. For better understanding of the subject, it is necessary to have clarity of sequence of events leading to disability and handicap.

The sequence is described as follows:

Disease → Impairment → Disability → Handicap

Disease is a condition of the body or some part or organ of the body in which its functions are disrupted or deranged.

Impairment is any loss or abnormality of psychological, physiological or anatomical structure or function.

**Annexure 1:** Selected list of institutions having a functional low vision unit in India [Indicative list]

Christian Medical College Ludhiana Punjab
Dr. R. P. Center for Ophthalmic Sciences Ansari Nagar New Delhi
Dr. Shroff Charity Eye Centre New Delhi
Venu Eye Institute and Research Centre New Delhi
St. Stephens Hospital Delhi
RIO Allahabad Uttar Pradesh
Tara Bai Desai Eye Hospital Jodhpur Rajasthan
SEWA Rural Jhagadia District Bharuch Gujarat
Blind People Association Ahmedabad Gujarat
Aso-Palov Eye Hospital Ahmedabad Gujarat
Gomabai Netrayala Neemuch Madhya Pradesh
Dhamtari Christian Hospital Dhamtari Chhattisgarh
Regional Institute of Ophthalmology Kolkata West Bengal
Viveka Nand Mission Hospital Haldia West Bengal
Sri Sankaradeva Netralaya Guwahati Assam
KBHB Hospita Mumbai Maharashtra
Lotus School of Optometry Mumbai Maharashtra
PBMS HV Desai Hospital Pune Maharashtra
Suraj Eye Institute Nagpur Maharashtra
RIO and Sarojini Devi Eye Hospital Hyderabad, Andhra Pradesh
LV Prasad Eye Institute Hyderabad Andhra Pradesh
Sankar Foundation Eye Hospital Visakhapatnam Andhra Pradesh
Regional Institute of Ophthalmology Chennai Tamil Nadu
Aravind Eye Hospital Madurai Tamil Nadu
Sankara Eye Centre Coimbatore Tamil Nadu
C.S.I. Kalyani Multi Specialty Hospital Chennai Tamil Nadu
Shankar Netralaya Chennai Tamil Nadu
Bejan Singh Eye Hospital Nagercoil Tamil Nadu
Institute of Ophthalmology and Joseph Hospital Tiruchirapalli Tamil Nadu
Josef Eye Hospital Trichi Kerela
Amrita Institute of Medical Sciences and Research Centre Kochi Kerala
National Association for the Blind selective state branches offers assistance related to low vision

Disability is any restriction or lack (resulting from impairment) of ability to perform an activity in the manner or within the range considered normal for a human being.

Handicap is a disadvantage for a given individual resulting from an impairment or disability that limits or prevents the fulfillment of a role that is normal (depending on age, sex, social and cultural factors) for that individual. The concept of handicap also includes the role of the society in creating barriers and limiting opportunities for people with disabilities.

## Rehabilitation

Rehabilitation involves combined and coordinated use of medical, social, educational, and vocational measures for training or retraining the individual to the highest possible level of functional ability. The three main strategies for rehabilitation of disabled are institution-based, outreach, and community-based.[5,6]

In general, rehabilitation encompasses the following:[7,8]

- Early detection, diagnosis, and intervention
- Improve, facilitate, stimulate and/or provide services for people with disabilities, their families and attendant
- Medical rehabilitation i.e., management of curable disability and lessening the disability to the extent possible
- Social, psychological, and other types of counseling and assistance
- Training in self-care activities including social graces, etiquette, mobility, communication, and daily living skills with special provisions as needed
- Provision of technical, mobility and other devices
- Specialized education services
- Vocational rehabilitation services including vocational guidance, training, open placement, and self-employment
- Certification of degree of disability and provision of available concessions/benefits
- Community awareness, advocacy, empowerment
- Follow-up

## Indian perspective for welfare of the disabled

The Constitution of India ensures equality, freedom, justice, and dignity of all individuals and implicitly mandates an inclusive society for all including persons with disabilities (PWDs). In the recent years, there have been vast and positive changes in the perception of the society towards PWDs. It has been realized that a majority of PWDs can lead a better quality of life if they have equal opportunities and effective access to rehabilitation measures. According to the Census 2001, there are 2.19 crore PWDs in India constituting 2.13% of the total population. This includes people with visual, hearing, speech, locomotor, and mental disabilities. Out of these, 75% live in rural areas, 49% are literate, and only 34% are employed. PWDs in India are defined as people who are suffering from not less than 40% of any ability as certified by medical authority.[9]

### Legislative, policy, and institutional framework[10]

The core of public health depends on law and science and it is also true that without the coercive power of the state, public health and modern society would be impossible. Law has to prohibit individuals who create situations for suffering for others. For this reason, public health must maintain the balance between individual autonomy and community protection. The public health actions are not intended to punish but to improve and monitor the health status in the community. To achieve the fundamental goals of protection, promotion and growth of individuals, groups and vulnerable population, various legislation and policies are drafted. Policy is a system that provides logical framework and rationality of decision making for the achievement of intended objectives. In short, policies set priorities and guide allocation of appropriate resources through establishment of an institutional framework.[11]

- GOI has enacted three legislations for PWDs viz.
  - National Trust for Welfare of Persons with Autism, Cerebral Palsy, Mental Retardation and Multiple Disability Act, 1999.
  - Persons with Disability (Equal Opportunities, Protection of Rights and Full Participation) Act, 1995.
  - Rehabilitation Council of India Act, 1992.
- A policy, National policy for people with disability, 2004.
- Seven national levels institutes viz.
  - National institute for the visually handicapped, Dehradun
  - National institute for the hearing handicapped, Mumbai
  - National institute for the mentally handicapped, Secunderabad
  - National institute for the orthopedically handicapped, Kolkata
  - National institute of rehabilitation training and research, Cuttack
  - Institute for the physically handicapped, New Delhi
  - National institute for the empowerment with persons with multiple disabilities, Chennai
- Five, Composite regional centers for PWDs
- Regional rehabilitation centers for persons with spinal injuries
- Indian spinal injury center, New Delhi
- Artificial limbs manufacturing corporation of India, Kanpur
- District disability rehabilitation centers

### Welfare programme/schemes for the rehabilitation of disabled in India[12–14]

The allocation of work related to medical component i.e., preventative, promotive, curative aspect is primarily planned, organized, and implemented by the Union Ministry of Health and Family Welfare, whereas rehabilitation is operated primarily by the nodal agency Union Ministry of Social Justice and Empowerment and administered by States/Union territories (UTs) through a number of stakeholders including ministries and department of Central/State Governments/UTs, Panchayati Raj Institutions (PRIs), Non governmental organizations (NGOs), disabled people organizations, advocacy groups, family and legal associations, experts and professionals for the welfare of disabled persons in the country. The program/schemes as proposed in Union Ministry of Social Justice and Empowerment are as follows:

#### Deendayal disabled rehabilitation scheme

The scheme provides support to the NGOs to deliver various rehabilitation services to people with disabilities. This includes support to schools for children with orthopedic, speech, hearing, visual and mental disabilities; vocational training centers to provide basic skills to PWDs; community-based rehabilitation program; half-way homes for psychosocial rehabilitation of treated and controlled mentally ill people; pre-school and early intervention programs; human resource development; support for setting up Braille presses; and placement services.

#### Scheme of assistance to disabled persons for purchase/fitting of aids/appliances (ADIP scheme)

The objective of this scheme is to assist the needy physically handicapped people with durable, modern, and standard aids and appliances.

#### Schemes for national scholarship of persons with disabilities

Every year 500 new scholarship are awarded for pursuing postmatric professional and technical courses of atleast one year duration under the scheme of national scholarship for PWDs. However, with respect to the students with cerebral palsy, mental retardation, multiple disabilities, and profound or severe hearing impairment, scholarship are awarded for pursuing studies from IX standard onwards. Students with 40% or more disability whose monthly family income does not exceed Rs. 15,000/- are eligible for scholarship. A scholarship of Rs 700/- per month to day-scholars and Rs. 1000/- per month to hostlers is provided to the students pursuing graduate and post-graduate level technical or professional courses. A scholarship of Rs. 400/- and Rs. 700/- per month to hostlers is provided for pursuing diploma and certificate level professional courses. In addition to the scholarship, the students are reimbursed the course fee subject to a ceiling of Rs. 10,000/- per year.

#### National awards for the welfare of persons with disabilities

The national awards are conferred not only to individuals and organizations that are working for the welfare of the PWDs but also on PWDs having outstanding achievements. The awards are given away on 3^rd^ December every year, which has been declared as International Day of Disabled Persons.

#### National handicapped finance and development corporation

Economic empowerment of disable persons is promoted for self-employment ventures, extend loans to PWDs for upgradation of their entrepreneurial skill, pursuing professional/technical education, and to assist self-employed individuals with disabilities in marketing their finished goods.

#### Science and technology project in mission mode

This project is engaged in development of technology which ultimately leads to a suitable device which is of high quality, durable, comfortable, and integrates the disabled into the mainstream of society. Funding is made available to established research and development centers, academic institutions, public sector industries, agencies undertaking research activities for the benefit of PWDs.

### Concession offered by the Government of India to blind persons[15,16]

According to guidelines by the Ministry of Social Justice and Empowerment, GOI, the minimum degree of disability should be 40% for an individual to be eligible for any concessions or benefits. State government also extends various benefits in addition to those initiated by the GOI.

#### Travel

The blind person traveling alone or with an escort on production of a certificate from a government doctor or a registered medical practitioner is eligible to get a concession to the tune of 75% if traveling in first, second or sleeper class in Indian railways. The Indian Airlines Corporation allows 50% concessional fare to blind persons on single journey or single fare round trip journey on all domestic flights. Escorts are to pay full fare however an air hostess/steward will look after the blind person not accompanied by escorts in flight.

#### Postage

There are no postal charges levied on transmission of blind literature packets [papers, periodicals, books printed in Braille, sound records, disc films, tapes and wires for the use of the blind and when sent by, or addressed to, an officially recognized institution for the blind] to inland/foreign destination if sent by surface route weighing upto 7 kg only. If packets are to be sent by air, prescribed airmail charges needs to be paid.

#### Telecommunication

The blind person is entitled for rental rebate of 50% on telephone facility on Non-OYT [s] category only. An educated unemployed handicap person with atleast VIII^th^ or middle schools pass for rural areas and matriculation or high school for urban areas is eligible for allotment of STD/PCO on priority basis.

#### Customs concessions

Selected goods when imported into India by a disable person for their personal use are exempted from customs duty if a certificate from a government medical officer/institution certifies that import of goods is essential to overcome the said disability.

#### Conveyance allowances

All central government employees who are in a regular establishment and who are blind or orthopaedically handicap are to be granted conveyance allowance at 5% of the basic pay subject to a maximum of Rs. 100/month.

#### Children education allowances

Grant of children educational allowances, reimbursement of tuition fees to central government employees are governed by central civil services orders.

#### Scheme of integrated education for disabled children

This is centrally sponsored scheme and was launched in 1974 by the then department of Social welfare. This scheme has however been transferred to the department of Education since 1982. Under the scheme, handicapped children are sought to be integrated in the normal school system. Financial assistance to the tune of 100% is provided to States/UTs for education of children suffering from certain mild handicaps in common schools with the help of necessary aids, incentives and specially trained teachers. The following types of disabled children are covered under this scheme: (a) children with locomotor handicaps; (b) mildly and moderately hearing impaired; (c) partially sighted children; (d) mentally handicapped educable group (IQ 50-70); (e) children with multiple handicap; (f) children with learning disabilities.

#### Income tax concessions

Section 80 DD provides for a deduction in respect of the expenditure incurred by an individual or Hindu Undivided Family (HUF) resident in India on the medical treatment [including nursing], training and rehabilitation of handicapped dependants. Section 88 B provides for an additional rebate from net tax payable by a resident individual who has attained the age of 65 years.

#### Employment

3% of vacancies in government employment in Grade C and D post are reserved for people with disabilities, 1% each for persons suffering from blindness or low vision, hearing impairment and locomotor disability and cerebral palsy. The GOI has set up 47 special employment exchanges in different states for the visually handicapped. As per the decision of the GOI, it has been instructed that recaning of chairs in government offices should be done by a visually impaired person as far as possible when the volume of work requires a full time chair caner.

#### Economic assistance

Under the scheme of public sector banks for orphanages, women's homes and physically handicapped persons including blind the benefits of the differential rate of interest are available to physically handicapped persons as well as institutions working for the welfare of the handicapped. Under the integrated rural development programme (IRDP), 3% quota is earmarked for physically handicapped persons.

## Definition of blindness[17,18] and low vision

In India, different definitions of disability are introduced for various purposes and as such, they have been based on various criteria. No single standard exists in India in order to evaluate disability. In common parlance, different terms such as disabled, handicapped, crippled, physically challenged are used interchangeably.[19] Visual acuity as well as field of vision has been considered for deciding blindness and in India it refers to a condition where a person suffers from any of the following conditions, namely: Total absence of sight; or visual acuity not exceeding 6/60 or 20/200 (Snellen) after best correction in the better eye; limitation of the field of vision subtending an angle of 20 degree or worse.

### 

#### Low vision

A person who after treatment of standard refraction has a visual acuity in better eye of <20/60 to light perception; or a visual field of <10° from the point of fixation, however, and is potentially able to use vision for planning or execution of a task [WHO].[20] Many persons especially children who have a corrected visual acuity of less than 3/60 in the better eye have useful vision and benefit from Low Vision services.

#### One-eyed person

Generally, the impairment of 40% or more is considered a handicap. As percentage of impairment in the case of one-eyed person is only 30%, according to the approved definition in medical parlance, a person with one good eye is not a blind person [Table 1]. The Committee of the Ministry of Social Justice and Empowerment on Recommendation of Standard Definition of Disability recommended that one eye-eyed person should be excluded from the other categories of visual impairment so that facilities and concessions available to severely and profoundly visually impaired persons are not eroded. The committee, however, felt that loss of one eye would not be considered as a disqualification on medical grounds unless a particular post is of such a technical nature that it requires of a person to have the coordinated use.

**Table 1:** Categories of visual disability for compensation in India

Category	Better eye	Worse eye	Percentage impairment
0	6/9-6/18	6/24-6/36	20
1	<6/18-6/30	6/60 to nil	40
2	<6/60-4/60 or field of vision 10°-20°	3/60 to nil	75
3	<3/60-1/60 or field of vision 10°	FC at 1 feet to nil	100
4	FC* at 1 feet to nil or field of vision 10°	FC at 1 feet to nil	100
One-eye blind	20/20	FC at 1 feet to nil	30

* FC - Finger counting

## Evaluation and need assessment for low vision

Visual impairment in general affects four main functional areas: Orientation/mobility, communication, activities of daily living (ADL) and sustained near vision task. Early intervention and special education can balance the negative effects of visual impairment. In many cases environmental adaptations, vision training, follow up for ensuring compliance, coordinating with stakeholders, removing myth and misconception and counseling would help in empowering the individual and/or enhancing functional residual vision.

The effect of low vision is not same for all people and the following assessment needs to be compiled for each individual before embarking upon the decision of assistive devices:[21]

- Extent of vision: Near and distance visual acuity
- Size of the visual field [if relevant]
- Effect of light and glare
- Extent of recognition and naming of colors
- Extent to which contrast affects their activities
- Extent of use of vision for different activities and purpose in the environment
- Extent to which a person sees and recognizes an object depends, amongst other on: Familiarity of the object; light; size; distance; contrast; color; detail or simplicity of the object
- Age, socioeconomic conditions, literacy status, and level of motivation

## Special assistive devices for the visually impaired

Assistive devices for the visually impaired can be broadly divided into the following categories: Education, mobility, vocational, daily living devices, low vision devices, and psychological test for vocational assessment and training.[22,23]

### 

#### Education devices[24]

Braille duplicators and writers, for example, Brailler and thermoform machine to convert material into Braille; Writing devices: Braille slates, Taylor postcard frame, pocket Braille frame; Braille paper; talking books and tape recorders: Material recorded on cassettes has emerged as the most popular mode of imparting education; Reading machines: Kurzweil reading machine, which reads typeset or typewritten text and turns it into speech; Braille computers: Braille Windows, Index Braille, Braille'n speak helps individual while working with personal computers; Mathematical devices: The Taylor arithmetic frame, abacus, talking calculator, spur wheel helps in learning mathematics; Geography and science devices: Sensory quill and three-dimensional raised maps help in learning geography, human physiology, zoology, and botany.

#### Mobility devices

Canes (symbol canes; guide canes; long canes; electronic travel devices), mobility show-card, mini beeper.

#### Vocational devices

Goniometer, attachment to lathe, spot welding, continuity tester, Braille micrometer.

#### Daily living devices

Daily living devices can be further classified into five broad categories namely, clocks and watches, games and puzzles, sports, kitchen equipment and personnel devices.

#### Low vision devices

Low Vision devices can be further divided into two types: Optical devices, which use lenses to magnify objects and non-optical devices and techniques, which make objects easier to use. A third category is electronic magnifier which is sometimes subsumed under non-optical devices. These devices include telescopes (telescopic spectacles, hand held, tele-bifocal spectacles), visualtek, schmidt reader, magnifying lenses (fixed focus; variable focus stand; half cylindrical rod; hand magnifier; folding; high plus spectacle; half eye spectacle-prism glasses; clip on magnifier), microscopic spectacles, visolett, fluorescent reading lamps, tinted lenses. Electronic magnifier/adaptive technology in the form of closed circuit television (CCTV), computer software (JAWS, MAGIC, text Braille software), speech synthesizer, talking books, overhead projector.[25–28]

Psychological assessment tests and training program is designed to develop a person's skill potential to the extent possible. These include Minnesota rate of manipulation test; Pennsylvania bi-manual work sample; Purdue pegboard; Crawford small parts dexterity test; Stanford-kolhs block design test for the blind; Blind learning aptitude test.

## New initiative on low vision services in approved 11^th^ five-year (2007-12) plan under NPCB

Strengthening Low Vision service is one of the thrust areas under 11^th^ five-year plan under NPCB in addition to ongoing activities. Regional Institutes of Ophthalmology (RIO) and government medical colleges are being developed as Low Vision units in a phased manner. Financial assistance for Low Vision devices like high plus spectacles, hand held magnifiers, stand magnifiers, telescope, video magnifiers [closed circuit television], absorptive lenses; field expanding devices are being provided by NPCB especially for poor patients. Eye surgeons working in public sector are being provided seven days orientation training on Low Vision services and financial support is borne by the GOI. Technical guidelines and comprehensive resource on Low Vision services is being developed involving all stakeholders for reference and dissemination. Improving quality of life of persons suffering from visual impairment involve patience, perseverance, multi-disciplinary approach with efficient coordination amongst stakeholders including medical, paramedical, social/psychological and educational professional. The gap between need and availability of Low Vision services is known globally, however, a beginning has been made by the GOI to address this issue and fruitful results will be evident in times to come.
